# Tribendimidine and Albendazole for Treating Soil-Transmitted Helminths, *Strongyloides stercoralis* and *Taenia* spp.: Open-Label Randomized Trial

**DOI:** 10.1371/journal.pntd.0000322

**Published:** 2008-10-15

**Authors:** Peter Steinmann, Xiao-Nong Zhou, Zun-Wei Du, Jin-Yong Jiang, Shu-Hua Xiao, Zhong-Xing Wu, Hui Zhou, Jürg Utzinger

**Affiliations:** 1 Department of Public Health and Epidemiology, Swiss Tropical Institute, Basel, Switzerland; 2 National Institute of Parasitic Diseases, Chinese Center for Disease Control and Prevention, Shanghai, People's Republic of China; 3 Helminthiasis Division, Yunnan Institute of Parasitic Diseases, Simao, People's Republic of China; 4 Jiangsu Institute of Parasitic Diseases, Wuxi, People's Republic of China; New York University School of Medicine, United States of America

## Abstract

**Background:**

Tribendimidine is an anthelminthic drug with a broad spectrum of activity. In 2004 the drug was approved by Chinese authorities for human use. The efficacy of tribendimidine against soil-transmitted helminths (*Ascaris lumbricoides*, hookworm, and *Trichuris trichiura*) has been established, and new laboratory investigations point to activity against cestodes and *Strongyloides ratti*.

**Methodology/Principal Findings:**

In an open-label randomized trial, the safety and efficacy of a single oral dose of albendazole or tribendimidine (both drugs administered at 200 mg for 5- to 14-year-old children, and 400 mg for individuals ≥15 years) against soil-transmitted helminths, *Strongyloides stercoralis*, and *Taenia* spp. were assessed in a village in Yunnan province, People's Republic of China. The analysis was on a per-protocol basis and the trial is registered with controlled-trials.com (number ISRCTN01779485). Both albendazole and tribendimidine were highly efficacious against *A. lumbricoides* and, moderately, against hookworm. The efficacy against *T. trichiura* was low. Among 57 individuals who received tribendimidine, the prevalence of *S. stercoralis* was reduced from 19.3% to 8.8% (observed cure rate 54.5%, *p* = 0.107), and that of *Taenia* spp. from 26.3% to 8.8% (observed cure rate 66.7%, *p* = 0.014). Similar prevalence reductions were noted among the 66 albendazole recipients. Taking into account “new” infections discovered at treatment evaluation, which were most likely missed pre-treatment due to the lack of sensitivity of available diagnostic approaches, the difference between the drug-specific net *Taenia* spp. cure rates was highly significant in favor of tribendimidine (*p* = 0.001). No significant adverse events of either drug were observed.

**Conclusions/Significance:**

Our results suggest that single-dose oral tribendimidine can be employed in settings with extensive intestinal polyparasitism, and its efficacy against *A. lumbricoides* and hookworm was confirmed. The promising results obtained with tribendimidine against *S. stercoralis* and *Taenia* spp. warrant further investigations. In a next step, multiple-dose schedules should be evaluated.

## Author Summary

More than a billion people are infected with intestinal worms and, in the developing world, many individuals harbor several kinds of worms concurrently. There are only a handful of drugs available for treatment, and drug efficacy varies according to the worm species. We compared the efficacy of a single oral dose of tribendimidine, a new broad-spectrum worm drug from China, with the standard drug albendazole for the treatment of hookworm, large roundworm (*Ascaris lumbricoides*), whipworm (*Trichuris trichiura*) and, for the first time, *Strongyloides stercoralis* and tapeworm (*Taenia* spp.). Our single-blind randomized trial was conducted in a village in Yunnan province, southwest China. Both drugs showed high efficacy against *A. lumbricoides* and a moderate efficacy against hookworm. Among 57 tribendimidine recipients, the prevalence of *S. stercoralis* was reduced from 19.3% to 8.8%, and that of *Taenia* spp. from 26.3% to 8.8%. Similar prevalence reductions were noted among the 66 albendazole recipients. Taking into account additional infections only discovered at treatment evaluation, the difference between the drug-specific *Taenia* spp. net cure rates was highly significant in favor of tribendimidine. In view of our promising results, multiple-dose schedules with tribendimidine against *S. stercoralis* and *Taenia* spp. should be evaluated next.

## Introduction

There is a growing awareness of the intolerable burden due to the so-called neglected tropical diseases [1]. Hence, new initiatives are underway for their control [2]. For helminth infections, the mainstay of control in high-burden areas rests on regular administration of anthelminthic drugs [1]–[5]. However, only a handful of drugs that have been developed many years ago are available [6], and there is considerable concern that resistance might develop, e.g., following repeated exposure of helminths to sub-curative doses. Reduced efficacy of common anthelminthic drugs is a major problem in veterinary medicine already, but for humans, it is of no clinical relevance thus far [7]. Another issue is that in a world where intestinal polyparasitism is common, yet neglected [8]–[12], and frequently used treatment regimens are only effective against a limited number of helminths [6], some species which are not effectively controlled by common drugs might increase in relative frequency, e.g., *Strongyloides stercoralis* and *Taenia* spp.

High prevalences of soil-transmitted helminths (*Ascaris lumbricoides*, hookworm, and *Trichuris trichiura*) and, consequently, a high level of intestinal multiparasitism, have recently been described from Nongyang, a settlement in Manguo administrative village, located in southwest Yunnan province, People's Republic of China [13]. Whilst *S. stercoralis* is endemic in the People's Republic of China [14], the local epidemiology of this parasite is not well understood. A prevalence of ∼15% was found in Nongyang [15]. Taeniasis and infections with the larval stage of *Taenia solium* that causes cysticercosis, have been documented throughout the People's Republic of China. Most infections occur in counties inhabited by non-Han nationalities whose traditional diets include the consumption of raw or undercooked meat [14],[16]. However, there is a paucity of epidemiologic data for taeniasis from the People's Republic of China, at least in the English literature [17]. In a recent cross-sectional survey, we found an egg-prevalence of *Taenia* spp. of 3.5% among 3220 individuals in Eryuan county, northwest Yunnan province [18]. In Nongyang, the prevalence of *Taenia* spp. was 5.1% [13].

The benzimidazoles, i.e., albendazole and mebendazole, are the most widely used drugs for the control of soil-transmitted helminthiasis [1],[19]. Both drugs have some effect against *S. stercoralis* and *Taenia* spp., but triple doses are recommended to achieve high cure rates [6],[20]. The drug of choice for treating *S. stercoralis* is ivermectin [21],[22]. Praziquantel and niclosamide are the recommended drugs against *Taenia* spp. [23]–[26]. Large-scale administration of ivermectin is the cornerstone of control programs targeting filarial infections, most notably onchocerciasis [27]. However, *Onchocerca volvulus* is not endemic in the People's Republic of China. Additionally, ivermectin is highly efficacious against *A. lumbricoides*, shows some activity against *T. trichiura*, but fails to cure hookworm infections. Since hookworms are common in the People's Republic of China, ivermectin is not commonly used for soil-transmitted helminth control in this country. These issues might explain why ivermectin is registered for human use in the People's Republic of China, but not readily available. Ivermectin, however, is produced at large scale for veterinary medicine, the bulk of which is exported.

Tribendimidine is an anthelminthic drug that has been registered in the People's Republic of China for use in humans [6],[28]. Tribendimidine is a symmetrical diamidine derivative of amidantel [28], its CAS registration number is 115103-15-6. Used at the current standard dose of 200 mg for children aged 5 to 14 years, and 400 mg for individuals aged ≥15 years, tribendimidine is safe and efficacious against *A. lumbricoides*, hookworm, and *Enterobius vermicularis* [28]. It also shows some activity against *T. trichiura* [29],[30], cestodes [28], and some trematodes [31],[32]. New research revealed in vitro and in vivo activity of tribendimidine against *Strongyloides ratti* [33].

The objective of this study was to assess the safety and efficacy of single-dose oral tribendimidine for treating intestinal helminth infections in a rural setting where polyparasitism is common, with a focus on the effects on *S. stercoralis* and *Taenia* spp. Comparison is made with single-dose oral albendazole as half of the participants were administered either drug. The primary outcome measures were the reduction of the infection prevalence of intestinal helminths, and the frequency and severity of adverse events. The secondary outcome measure was the Kato-Katz-derived egg count reduction of common soil-transmitted helminths. Multiple stool samples were collected before and after drug administration and examined by different diagnostic tools to enhance the diagnostic sensitivity.

## Materials and Methods

### Study site and population

The study was carried out in Nanweng, a village in Menghai county, Xishuangbanna prefecture, Yunnan province, People's Republic of China, from May to July 2007. Nanweng has 81 resident families and is situated on the slope of a mountain, 1650 m above sea level at 21.77 N latitude and 100.40 E longitude. The village is exclusively inhabited by the Bulang ethnic group. Its economic basis is provided by the surrounding tea plantations and the more distant, partially irrigated rice and other crop fields. Untreated tap water is delivered to every house but no sanitation facilities are available in the entire village.

### Field and laboratory procedures

The leader of the Menghai-based county Center for Disease Control and Prevention (CDC) briefed the village authorities about the study. Village leaders then informed the residents who were all invited to participate. Over a 3-week period, 20–30 families were enrolled weekly, and household as well as individual questionnaires that have been used before [15],[18] were administered. Children below the age of 15 years were assisted by their parents or legal guardians to answer the questions, and infants younger than 2 years were excluded from the study. Participants were asked to provide a large stool sample in pre-labeled collection containers. Filled containers were collected daily and exchanged by empty ones with the goal to obtain 3 stool samples per participant. The evaluation of the treatment efficacy commenced 2 weeks post-treatment and followed the same field procedures. Stool samples were collected over a 2-week period, again aiming to obtain 3 samples per study participant.

Stool samples were collected in the village between 07:00 and 09:00 a.m., transferred to the laboratory in Menghai city, and processed within a maximum of 6 hours of receipt. First, ∼10 g of stool were placed on a gauze which was embedded on a wire mesh in a glass funnel equipped with a sealable rubber tube, the so-called Baermann device [33]. The funnel was then filled with de-ionized water and illuminated from below with an incandescent bulb. Second, for the Koga agar plate test [34], ∼2 g of stool were placed in the centre of a 9 cm Petri dish with freshly prepared nutrient agar. Third, a single 41.7 mg Kato-Katz thick smear [35] was prepared on a microscope slide and helminth eggs were enumerated after a clearing time of 30–60 min.

The lowest 45 ml of the liquid in the Baermann funnel were drained after 2 h, centrifuged, and the sediment was examined for *S. stercoralis* larvae at low magnification (40×). Koga agar plates were inspected for helminth larvae at similar magnification after a 2-day incubation period at 28°C in a humid chamber. Subsequently, all plates were rinsed with 12 ml sodium acetate–acetic acid–formaline (SAF) solution [36], gently scraped, and the eluent was centrifuged. The sediment was examined for helminth larvae, i.e., *S. stercoralis* and hookworms. Helminth larvae were differentiated based on established anatomical criteria, i.e., considering the buccal cavity and genital primordium of first-stage (L_1_) *S. stercoralis* larvae (buccal cavity: short; genital primordium: prominent), and the tip of the tail of third-stage (L_3_) *S. stercoralis* larvae (tip of the tail: cut). Samples were considered positive if larvae were detected at any stage and by any test. Helminth eggs in the Baermann and Koga sediment were also noted.

### Randomization and drug administration

Study participants who had submitted at least 1 stool sample were listed according to their identification number in ascending order. Subsequently, a random sequence of 0's and 1's was generated in Excel (Microsoft Corp.) by the study coordinator, and aligned with the list of eligible study participants. Individuals matched with a “0” were assigned albendazole, whereas those with a “1” were assigned tribendimidine. Albendazole and tribendimidine were purchased from Shanxi Hanwang Medicine Co. Ltd. (Han Zhong, People's Republic of China) and Shandong Xinhua Pharmaceutical Co. Ltd. (Zibo, People's Republic of China), respectively. Drugs were administered as single 200 mg oral dose for children aged 5 to 14 years, and 400 mg for individuals ≥15 years of age. Drugs at the appropriate dosage according to participants' age were packed in identical envelopes labeled with the participant's name only. Hence, our study was an open-label trial, i.e., participants did not know which drug they received. Participants were asked to avoid alcohol consumption on the day of drug administration. After dinner time, teams of fieldworkers visited the village, met the participants at home and asked them about signs of acute or chronic illness, and alcohol consumption. Women aged ≥14 years were asked about pregnancy. Drugs were handed out together with fresh bottled water to healthy, non-drunk and non-pregnant participants, and drug intake was observed. Those treated were asked to report any potential drug-related signs or symptoms including sleeping troubles to the accompanying medical doctor.

### Ethical considerations and end-of-study treatment

The study was approved by the institutional research commission of the Swiss Tropical Institute (Basel, Switzerland). Ethical clearance was obtained from the Ethics Committee of Basel (EKBB), Switzerland (reference no. 149/07) and the Ethics Committee of the National Institute of Parasitic Diseases, Chinese Center for Disease Control and Prevention (Shanghai, People's Republic of China). The trial is registered with controlled-trials.com under the registration number ISRCTN01779485. The study procedures, potential risks, and benefits were explained to the village leaders. After their consent to perform the study, field workers visited the homes of the selected families where detailed information was provided to all potential participants, and questions were answered. Emphasis was placed on voluntary participation and the option to quit the study at any moment without further obligation. Written confirmation that full information had been provided and individual participation was voluntary (informed consent) was obtained from the head of each participating household or a literary substitute (adult child or relative), and this procedure was approved by the above-mentioned ethical committees.

A single 200 mg (for children aged 5 to 14 years), or 400 mg (individuals aged ≥15 years) oral dose of albendazole was offered to those participants who were not eligible for randomization because they had failed to provide any stool sample. The assessment of their health status and the treatment procedures followed the same protocol as for study participants, but the treatment outcome was not assessed.

Locally-used remedies for *Taenia* spp. infection and ivermectin for treating *S. stercoralis* at the standard dose (200 µg/kg) were provided at the end-of-study follow-up. Finally, albendazole was provided to the village authorities for later distribution to untreated inhabitants and participants who still harbored active infections.

### Data management and statistical analysis

The target sample size was 130 individuals, based on the following assumptions: Prevalence of *S. stercoralis*: 20%; efficacy of tribendimidine and albendazole against *S. stercoralis*: 85% (similar to ivermectin at standard dosage [37]) and 0%, respectively, with a confidence level of 95% and a power of 80%.

**Figure 1:**
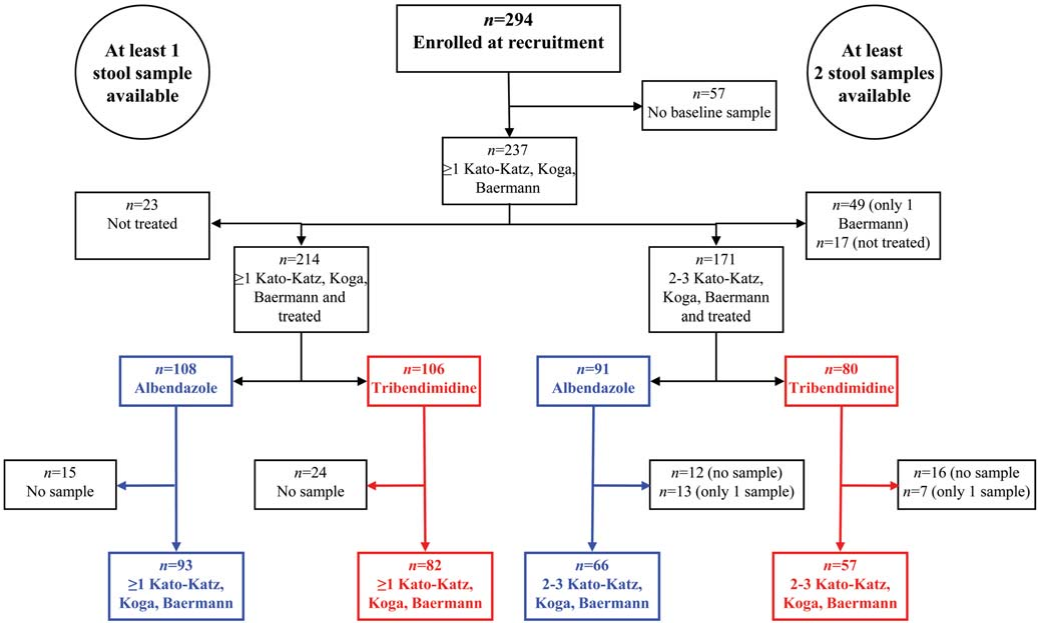
Participation and stool sample submission compliance in a community-based open label efficacy study involving repeated stool sample collection and anthelminthic treatment by either albendazole or tribendimidine; Nanweng village, Yunnan province, People's Republic of China.

The questionnaire data were double-entered and cross-checked in EpiData version 3.1 (EpiData Association; Odense, Denmark). The laboratory data were examined for internal consistency, and merged with the questionnaire data. Statistical analysis was done with STATA version 9.2 (StataCorp; College Station, USA). Our final study cohort consisted of individuals aged ≥5 years who had submitted at least 2 stool samples at baseline, did not suffer from any chronic or acute illness, had not drunk alcohol on the day of drug administration, for women were not pregnant, had taken the randomly assigned drug, and had again at least 2 stool samples examined at the end-of-study survey. Thus, statistical analyses only considered treated individuals with complete diagnostic data records, stratified by drug (see Figure 1, end points of the right arm). Multiple stool readings at baseline and follow-up were required in order to boost diagnostic sensitivity [13],[15].

The infection status was determined based on the pooled results from the different diagnostic methods (i.e., soil-transmitted helminths and *Taenia* spp.: all tests; *S. stercoralis*: Baermann and Koga agar plate tests). Pearson's χ^2^-test and Fisher's exact test, as appropriate, were used to assess the association between infection and demographic variables. Treatment outcomes by drug and the differences between albendazole and tribendimidine were explored by calculating drug-specific prevalence reductions, and analyzing the difference between the observed cure rates (2-sample test of proportions). The infection intensity for the common soil-transmitted helminths was determined based on the quantitative Kato-Katz thick smear readings, multiplied by a factor of 24. Infection intensities for individual study participants were obtained by calculating the arithmetic mean of the available egg counts. The arithmetic mean of the averaged egg counts among the infected yielded a summery measure of infection intensity among the infected population. The individual infection intensities were subsequently stratified according to the classification proposed by the World Health Organization (WHO) [38]. The effects of albendazole and tribendimidine on the infection intensities in the respective groups were analyzed using a paired *t*-test, and drug-specific infection intensity reductions were compared by a 2-sample test of proportions.

## Results

### Participation

We counted 294 family members, aged above 2 years, in the 81 resident families. Another 60 individuals were recorded in the village registry but they had either left the village, were younger than 2 years, or refused to answer the questionnaire. Figure 1 shows that 106 (36%) of the eligible participants provided none (*n* = 57), or only a single (*n* = 49) stool sample of sufficient quantity to perform all diagnostic tests. The remaining 188 individuals (64%) were 5 to 87-year-old and among them, 17 could not be treated due to pregnancy (*n* = 8), ill health (*n* = 5) or other reasons (*n* = 4). The randomization of the 171 participants with complete parasitological baseline data who were eligible for treatment resulted in equal-sized groups for single-dose oral tribendimidine or albendazole administration, but stool sample submission at baseline and actual treatment rates were somewhat lower among tribendimidine recipients.

Of the 91 participants treated with albendazole, 2 to 3 sufficiently large stool samples were available from 66 individuals (73%) at the end-of-study follow-up. A similar stool sample submission rate was observed for the 80 tribendimidine recipients (71%). The final cohort consisted of 123 individuals who received either albendazole (*n* = 66) or tribendimidine (*n* = 57), and submitted at least 4 (2 pre- plus 2 post-treatment) sufficiently large stool samples to carry out the full range of diagnostic tests. While drop-out rates were similar for males and females (42.2% *versus* 41.5%), full participation ranged from 21% for 10 to 14-year-olds to 58% for those aged ≥40 years (data not shown).

### Helminth infections at baseline

Considering the joint results of the 2 to 3 Kato-Katz, Koga agar plate and Baermann tests, our final cohort showed high prevalences of *T. trichiura* (87.8%), hookworm (74.8%), and *A. lumbricoides* (72.4%). The prevalences of *Taenia* spp. and *S. stercoralis* were 26.0% and 17.9%, respectively.

Table 1 shows that *Taenia* spp. infections were significantly more prevalent among males (33.9%) than females (18.0%, χ^2^ = 4.01, degrees of freedom (df) = 1, *p* = 0.045) and increased with age, albeit not significantly (*p* = 0.163). An increase with age was also noted in the prevalence of *S. stercoralis* infections. No *S. stercoralis* were diagnosed among pre-school children and students as opposed to farmers (*p* = 0.121); *Taenia* spp. was also more common among farmers (27.3%) than pre-school children and students (8.3%, *p* = 0.292). Illiterates had a lower *A. lumbricoides* prevalence than those with formal education (50.0% vs. 79.2%, χ^2^ = 9.32, df = 1, *p* = 0.002), reflecting the generally lower prevalence among older age groups. Table 1 also summarizes the infection intensities for the common soil-transmitted helminths according to the Kato-Katz results. While most hookworm and *T. trichiura* infections were of light intensity, a considerable number of moderate and a few heavy infections were noted for *A. lumbricoides*.

**Table 1:** Baseline prevalence of *S. stercoralis*, *Taenia* spp., *A. lumbricoides*, *T. trichiura* and hookworm infections, and Kato-Katz test-derived infection intensities, among 123 study participants from Nanweng village, Yunnan province, People's Republic of China who had submitted at least 2 stool samples before and after treatment with either albendazole or tribendimidine, stratified by demographic variables.

	*n*	*S. stercoralis*			*Taenia* spp.			*A. lumbricoides*			Hookworm			*T. trichiura*		
		Pos.	% (95% CI)	χ^2^ (*p* value)	Pos.	% (95% CI)	χ^2^ (*p* value)	Pos.	% (95% CI)	χ^2^ (*p* value)	Pos.	% (95% CI)	χ^2^ (*p* value)	Pos.	% (95% CI)	χ^2^ (*p* value)
Total	123	22	17.9 (11.0, 24.8)	n.a.	32	26.0 (18.2, 33.9)	n.a.	89	72.4 (64.3, 80.4)	n.a.	92	74.8 (67.0, 82.6)	n.a.	108	87.8 (81.9–93.7)	n.a.
Sex: Female	61	10	16.4	0.18 (0.668)	11	18.0	4.01	49	80.3	3.84 (0.050)	47	77.1	0.33 (0.568)	57	93.4	3.59
Male	62	12	19.4		21	33.9	(0.045)	40	64.5		45	72.6		51	82.3	(0.058)
Age: 5–14 years	15	1	6.7	n.a. (0.493)^c^	1	6.7	n.a. (0.163)^c^	13	86.7	n.a. (0.138)^c^	11	73.3	n.a. (0.848)^c^	15	100	n.a. (0.451)^c^
15–24 years	13	1	7.7		2	15.4		12	92.3		10	76.9		11	84.6	
25–39 years	35	7	20.0		12	34.3		25	71.4		28	80.0		31	88.6	
≥40 years	60	13	21.7		17	28.3		39	65.0		43	71.7		51	85.0	
Education^a^																
Illiterate	34	9	26.5	n.a. (0.298)^c^	9	26.5	n.a. (0.821)^c^	17	50.0	9.32 (0.002)	24	70.6	0.409 (0.522)	28	82.4	0.50 (0.478)
Primary & middle school	72	12	16.7		21	29.2		57	79.2		55	76.4		63	87.5	
Occupation^b^																
Farmer	110	22	20.0	n.a. (0.121)^c^	30	27.3	n.a. (0.292)^c^	79	71.8	n.a. (0.510)^c^	83	75.5	n.a. (0.498)^c^	96	87.3	n.a. (0.356)
Pre-school & student	12	0	0		1	8.3		10	83.3		8	66.7		12	100	
Infection intensity^d^	123															
Mean; SE^e^		n.a.	n.a.	n.a.	n.a.	n.a.	n.a.	84	11810; 2184	n.a.	88	360; 83	n.a.	108	493; 72	n.a.
No		n.a.	n.a.	n.a.	n.a.	n.a.	n.a.	39	31.7	n.a.	35	28.5	n.a.	15	12.2	n.a.
Light								45	36.6		86	69.9		93	75.6	
Moderate								35	28.5		1	0.8		15	12.2	
Heavy^f^								4	3.3		1	0.8		0	0	

a Among the 106 individuals aged ≥18 years; educated group includes 2 participants with higher education (apprenticeship, college).

b 1 participant with other occupation than farmer (civil servant) excluded.

c Fisher's exact test.

d Eggs per gram of stool, according to Kato-Katz.

e Arithmetic mean among the infected; standard error (SE).

f According to the stratification put forward by WHO [38].

Pos., positive; n.a., not applicable.

### Efficacy of tribendimidine and albendazole

As detailed in Table 2, single-dose oral albendazole and tribendimidine significantly reduced the prevalence of *A. lumbricoides* (albendazole: from 75.8% to 0; tribendimidine: from 68.4% to 5.3%; both *p*<0.001), and hookworm (albendazole: from 69.7% to 21.2%; tribendimidine: from 80.7% to 38.6%; both *p*<0.001). Whilst the difference between the drug-specific cure rates against *A. lumbricoides* showed borderline significance in favor of albendazole, there was no difference in the efficacy of the two drugs against hookworm. Although neither albendazole nor tribendimidine resulted in a significant reduction in the prevalence of *T. trichiura*, single-dose oral albendazole was significantly more efficacious than tribendimidine in curing *T. trichiura* (*p* = 0.014). Single-dose albendazole and tribendimidine significantly reduced the arithmetic mean egg counts among those who were infected at baseline (all *p*<0.05) except for tribendimidine administered to individuals with *T. trichiura* (*p* = 0.136). Both drugs decreased the mean egg counts of *A. lumbricoides* and hookworm to a similar extent, but mean *T. trichiura* egg counts declined significantly more following albendazole than tribendimidine treatment (*p* = 0.005).

**Table 2:** Prevalence and cure rates of *A. lumbricoides*, *T. trichiura*, hookworm, *S. stercoralis*, and *Taenia* spp., and Kato-Katz-derived infection intensity reductions among 123 study participants from Nanweng village, Yunnan province, People's Republic of China who submitted at least 2 stool samples before and after treatment with single-dose oral albendazole or tribendimidine.

Drug	Albendazole										Tribendimidine									
Parasite	*A. lumbricoides*		*T. trichiura*		Hookworm		*S. stercoralis*		*Taenia* spp.		*A. lumbricoides*		*T. trichiura*		Hookworm		*S. stercoralis*		*Taenia* spp.	
	*n*	Prev.	*n*	Prev.	*n*	Prev.	*n*	Prev.	*n*	Prev.	*n*	Prev.	*n*	Prev.	*n*	Prev.	*n*	Prev.	*n*	Prev.
Total *n*	66										57									
Baseline: positive	50	75.8	60	90.9	46	69.7	11	16.7	17	25.8	39	68.4	48	84.2	46	80.7	11	19.3	15	26.3
Follow-up: still positive	0	0	53	80.3	14	21.2	7	10.6	7	10.6	3	5.3	48	84.2	22	38.6	5	8.8	5	8.8
Prevalence reduction	75.8 (65.5, 86.1)^a^ *p*<0.001		10.6 (−1.2, 22.4)^a^ *p* = 0.083		48.5 (33.7, 63.3)^a^ *p*<0.001		6.1 (−5.6, 17.8)^a^ *p* = 0.307		15.2 (2.3, 28.1)^a^ *p* = 0.024		63.1 (49.7, 76.5)^a^ *p*<0.001		0 (−13.4, 13.4)^a^ *p* = 1.000		42.1 (25.8, 58.4)^a^ *p*<0.001		10.5 (−2.1, 23.1)^a^ *p* = 0.107		17.5 (3.9, 31.1)^a^ *p* = 0.014	
Cured/cure rate	50	100	7	11.7	32	69.6	4	36.4	10	58.8	36	92.3	0	0	24	52.2	6	54.5	10	66.7
Difference drug-specific cure rates	7.7 (−0.7, 16.1)^a^ *p* = 0.046		11.7 (3.6, 19.8)^a^ *p* = 0.014		17.1 (−2.5, 36.7)^a^ *p* = 0.093		18.1 (−22.8, 59.0)^a^ *p* = 0.394		7.9 (−25.5, 41.3)^a^ *p* = 0.645		7.7 (−0.7, 16.1)^a^ *p* = 0.046		11.7 (3.6, 19.8)^a^ *p* = 0.014		17.1 (−2.5, 36.7)^a^ *p* = 0.093		18.1 (−22.8, 59.0)^a^ *p* = 0.394		7.9 (−25.5, 41.3)^a^ *p* = 0.645	
Inf. intensity baseline^b^																				
Mean; SE^c^	14415; 3498		540; 100		251; 58		n.a.		n.a.		8805; 2378		434; 105		462; 151		n.a.		n.a.	
Light	21	31.8	50	75.8	43	65.2	n.a.	n.a.	n.a.	n.a.	24	42.1	43	75.4	43	75.4	n.a.	n.a.a	n.a.	n.a.
Moderate	22	33.3	10	15.2	0	0					13	22.8	5	8.8	1	1.8				
Heavy^d^	2	3.0	0	0	0	0					2	3.5	0	0	1	1.8				
Inf. intensity follow-up^b^																				
Mean; SE^c^	0; n.a.		256; 50		121; 44		n.a.		n.a.		2128; 1996		299; 44		118; 37		n.a.		n.a.	
Light	0	0	49	74.2	11	16.7	n.a.	n.a.	n.a.	n.a.	2	3.5	48	84.2	21	36.8	n.a.	n.a.	n.a.	n.a.
Moderate	0	0	3	4.6	0	0					1	1.8	2	3.5	0	0				
Heavy^d^	0	0	0	0	0	0					0	0	0	0	0	0				
Inf. Intensity reduction^e^																				
Mean; SE^c^	14415; 3498		318; 82		220; 54		n.a.		n.a.		8642; 2386		138; 91		408; 146		n.a.		n.a.	
*t*; *p* value	4.12; <0.001		3.89; <0.001		4.03; <0.001		n.a.		n.a.		3.62; <0.001		1.52; 0.136		2.79; 0.008		n.a.		n.a.	
Difference drug-specific inf. intensity reductions (%)	1.9 (−2.4, 6.2)^a^ *p* = 0.353		27.1 (9.0, 45.2)^a^ *p* = 0.005		0.7 (−12.9, 14.3)^a^ *p* = 0.920		n.a.		n.a.		1.9 (−2.4, 6.2)^a^ *p* = 0.353		27.1 (9.0, 45.2)^a^ *p* = 0.005		0.7 (−12.9, 14.3)^a^ *p* = 0.920		n.a.		n.a.	

a 95% confidence interval.

b Eggs per gram of stool among infected, according to Kato-Katz.

c Arithmetic mean; standard error (SE).

d According to the stratification put forward by WHO [38].

e Reduction of the arithmetic mean number of eggs per gram of stool among the infected at baseline.

Inf., infection; n.a., not applicable; Prev., prevalence.

Single-dose albendazole and tribendimidine resulted in prevalence reductions of *S. stercoralis* of 6.1% and 10.5%, respectively, which was not statistically significant (both *p*>0.05). The prevalence of *Taenia* spp. was reduced from 25.8% to 10.6% after albendazole administration (*p* = 0.024), whilst among the tribendimidine recipients, the prevalence was lowered from 26.3% to 8.8% (*p* = 0.014). Table 2 also shows that after administration of albendazole, *S. stercoralis* larvae could still be found among 7 of the previously 11 infected individuals (observed cure rate: 36.4%). Among those treated with tribendimidine, 6 out of 11 individuals were larvae-free (observed cure rate: 54.5%; difference: 18.1%, *p* = 0.394). The baseline *Taenia* spp. prevalence of 25.8% and 26.3% among those given albendazole and tribendimidine was reduced by 58.8% and 66.7%, respectively (difference: 7.9%, *p* = 0.645).

Taking into account *S. stercoralis* and *Taenia* spp. infections that had only been recognized at treatment evaluation (these infections were most likely missed pre-treatment due to the lack of diagnostic sensitivity of the available tests), the efficacy of the drugs was lower (Table 3). In both treatment groups, *S. stercoralis* was diagnosed in 2 individuals previously declared uninfected. Hence, the net cure rate was 18.2% for albendazole and 36.4% for tribendimidine recipients (difference: 18.2%, *p* = 0.338). The number of “new” *Taenia* spp. infections in the albendazole group diagnosed during follow-up equaled the number of recoveries (*n* = 10), resulting in a zero overall cure rate. Among tribendimidine recipients, only 2 additional *Taenia* spp. infections were found; the prevalence reduction showed borderline significance (−14.0%, *p* = 0.058). The net cure rate of *Taenia* spp. in the tribendimidine recipients was 53.3%, significantly different from the albendazole group (difference: 53.3%, *p* = 0.001).

**Table 3:** Prevalence and net cure rates of *S. stercoralis* and *Taenia* spp. taking into account “new” infections discovered at follow-up among previously “negative” participants.

	*S. stercoralis*				*Taenia* spp.			
	Albendazole		Tribendimidine		Albendazole		Tribendimidine	
	n	Prev. (%)	n	Prev. (%)	n	Prev. (%)	n	Prev. (%)
Total samples	66	100	57	100	66	100	57	100
Positive before treatment	11	16.7/100	11	19.3/100	17	25.8/100	15	26.3/100
Still positive after treatment	7	10.6/63.6	5	8.8/45.5	7	10.6/41.2	5	8.8/33.3
“New” positive after treatment	2	3.0/18.2	2	3.5/18.2	10	15.2/58.8	2	3.5/13.3
Total positive after treatment	9	13.6/81.8	7	12.3/63.6	17	25.8/100	7	12.3/46.7
Net prevalence reduction	3.1 (−9.1–15.3)^a^ *p* = 0.619		7.0 (−6.3–20.3)^a^ *p* = 0.306		0.0 (−14.9–14.9)^a^ *p* = 1.000		14.0 (−0.3–28.3)^a^ *p* = 0.058	
Net cured/net cure rate	2	18.2	4	36.4	0	0	8	53.3
Difference between drug-specific net cure rates	18.2 (−18.2, 54.6)^a^ *p* = 0.338				53.3 (28.1, 78.5)^a^ *p* = 0.001			

Effect of single-dose oral albendazole or tribendimidine among 123 study participants from Nanweng village, Yunnan province, People's Republic of China who submitted at least 2 stool samples before and again after treatment (Prev., prevalence).

a 95% confidence interval.

### Adverse events

No adverse events were mentioned by participants treated with single-dose oral albendazole. In the tribendimidine group, an 87-year-old woman reported mild sleeping disorders, headache, dizziness, and gastrointestinal symptoms, including a single episode of vomiting. This subject had a light infection with *T. trichiura* and hookworm at baseline. Upon treatment evaluation, the participant did not submit any further stool samples, and hence was excluded from the final cohort.

## Discussion

To our knowledge, this is the first investigation assessing the safety and efficacy of single-dose tribendimidine for treating *S. stercoralis* and *Taenia* spp. infections, and the first clinically-monitored use of tribendimidine in a setting with high rates of intestinal multiparasitism. Indeed, infections with one of the three main soil-transmitted helminths were found in 72.4–87.8% of the study participants, and only 4 of the 123 individuals in our final cohort (3.3%) harbored none of these helminths. The prevalence of *S. stercoralis* and *Taenia* spp. at baseline was 17.9% and 26.0%, respectively. Our study was an open-label trial, comparing a single oral dose of albendazole with that of tribendimidine, with both drugs administered at either 200 mg or 400 mg according to participants' age.

The final study cohort comprised less than 50% of those initially contacted. Whilst the cohort had a similar sex distribution than the total population of Nanweng village, it was considerably biased toward older age groups. We screened multiple stool samples for intestinal helminths and randomly assigned the participants to either albendazole or tribendimidine. Treatment outcome was assessed 2 to 4 weeks after dosing using multiple stool samples and a diversity of diagnostic approaches. The prevalence of *S. stercoralis* was not significantly reduced by either albendazole or tribendimidine, and no significant drug-specific difference was observed. Among individuals infected with *Taenia* spp. at baseline, the observed cure rates of 58.8% for albendazole and 66.7% for tribendimidine showed statistical significance (both *p*<0.05). During follow-up, however, additional infections were found, mainly *Taenia* spp. among those who had received albendazole, and the difference between the drug-specific overall cure rates was 53.3% (*p* = 0.001). The observed cestocidal effect of tribendimidine, for the time being, should rather be regarded as an indication of a possible activity than as a proof-of-concept. This is due to the obvious diagnostic challenges encountered in field-based clinical studies on large cestodes.

In the current trial, albendazole generally performed slightly better than tribendimidine in curing common soil-transmitted helminth infections. The most notable difference was seen with *T. trichiura*, confirming earlier observations that single-dose albendazole is somewhat more efficacious than single-dose tribendimidine against this nematode [6]. The observed cure rates against hookworm and *T. trichiura* following single-dose albendazole are rather low compared to the results of a recent meta-analysis of this and other WHO-recommended anthelminthics commonly used against common soil-transmitted helminth infections [39]. We speculate that this observation is rather reflecting the rigorous diagnostic approach employed than an unusually low susceptibility of local hookworm and *T. trichiura* to albendazole. For example, hookworm infections could not only be detected by the widely used Kato-Katz technique, but also by the Koga agar plate method. However, the low cure rates observed in this study should also be seen as a warning sign and call for monitoring of drug efficacy and the potential emergence of drug resistance [38].

The inclusion of only 123 individuals who met our sample submission requirements into the final study cohort reduced the reported compliance rate but increased the reliability of the results due to the increased overall sensitivity of the employed diagnostic methods [15]. Indeed, a lower prevalence was found among those 175 participants who had at least 1 stool sample analyzed, but the drug-specific efficacies were similar (data not shown).

The discovery of notable numbers of infections among those who were deemed negative before treatment can be explained by at least 2 mechanisms, or a combination thereof. First, it is conceivable that the baseline evaluation fell within the prepatent period of recent infections. Second, it is well known that parasitological diagnosis of both *S. stercoralis* [40] and *Taenia* spp. [25],[41] lacks sensitivity. For *S. stercoralis*, the main remedy for this challenge is screening of multiple stool specimens [21], whilst for *Taenia* spp., sensitive coproantigen enzyme-linked immunosorbent assay (ELISA) tests provide valuable alternatives [23],[25],[26],[41]. The current diagnostic ‘gold’ standard to confirm treatment success in taeniasis is the recovery of the tapeworm scolex. Alternatively, the absence of proglottids from stools and underwear over a period of 3 months also provides solid proof of cure. However, such extensive observation is usually only feasible in hospital settings. Re-infection after treatment can almost certainly be excluded for *Taenia* spp., and it is rather unlikely for *S. stercoralis*. We speculate that in our study, the limited sensitivity of the diagnostic tools was more significant since the 3 to 5-week period between baseline and follow-up investigation is rather short for any notable level of re-infections.

We are confident that our results are valid despite the imperfect sensitivity of the employed diagnostic tests, not least due to our rigorous sampling effort. This assumption is supported by the following observations. For *S. stercoralis*, the numbers of “new” infections at follow-up was similar in both treatment groups (both *n* = 2), thus reducing the observed cure rate but not affecting the overall conclusion that both drugs exhibit some effect at the employed dosage. A mathematical model [42] for the prediction of “true” prevalence further suggested an underestimation of the *S. stercoralis* prevalence by the employed procedures within the range actually observed in the present trial [15]. After a study involving extensive stool sample collection and analysis by the Baermann technique, Dreyer and co-workers [43] suggested that at least 4 stool samples need to be collected to accurately assess the *S. stercoralis* infection status, and that only those with at least 2 positive test results should be included in clinical drug trials. In our study, we only included those individuals who had at least 2 stool specimens examined with 2 different diagnostic approaches. Thus, at least 4, and ideally 6, results were available to judge the infection status of the participants both before and after drug administration. Among the 22 *S. stercoralis* positives at baseline, 6, 6, 3, 5, 1 and 1 individuals had 1, 2, 3, 4, 5 and 6 positive test results, respectively. The 10 arguably cured individuals had 1 (*n* = 3), 2 (*n* = 3), 3 (*n* = 2) and 4 (*n* = 2) positive baseline test results. Our findings indicate that participants with only 1 or 2 positive tests at baseline were not more likely to be considered cured at treatment evaluation than those with multiple positive tests. However, infections were still found in all participants with 5 or 6 positive tests at baseline. The four individuals who were only found to be infected at treatment evaluation then had 1, 1, 2 and 3 positive test results. Finally, the observed activity of albendazole against *Taenia* spp. among those who were found to be infected at baseline has to be put into perspective with the high number of “newly” detected infections at treatment evaluation. Among tribendimidine recipients, only few additional *Taenia* spp. infections were found, indeed indicating that single-dose tribendimidine, but not albendazole, might have some effect against *Taenia* spp.

Unfortunately, the eggs of *T. saginata*, *T. solium*, and *T. asiatica* cannot be readily distinguished microscopically [23],[25]. Hence, we are not in a position to determine their relative frequency in our study population. However, the reported and observed diets suggest that the locally dominant species is *T. solium* or possibly *T. asiatica* since Bulang favor raw pork over raw beef.

As a next step, the efficacy of multiple-dose tribendimidine could be assessed as our results indicate some, albeit currently unsatisfactory effect of this drug against *S. stercoralis* and *Taenia* spp. In future studies with a focus on these 2 parasites rather than the common soil-transmitted helminths, the reference drug should be praziquantel or niclosamide for *Taenia* spp., and ivermectin for *S. stercoralis*. Alternatively, triple-dose albendazole might be used as reference treatment [20]. When further investigating the efficacy of tribendimidine against large cestodes, including *Taenia* spp. in humans, we propose to treat a small group of confirmed taeniasis cases who agreed to submit multiple stool samples and observe proglottids in their stools and underwear over extended time periods.

Infections with *A. lumbricoides* and hookworm are the main targets for single-dose mass chemotherapy using albendazole or mebendazole. Discussions are underway in the People's Republic of China for the larger-scale use of tribendimidine. Efficacy of the latter drug on other intestinal parasites would be of considerable public health significance, which is explained by the geographic overlap of different helminth infections, including *S. stercoralis* and *Taenia* spp. Treatment of individuals with multiple species parasite infections, including *S. stercoralis* and *Taenia* spp., is likely to occur. Hence, there is a pressing need to determine the most efficacious tribendimidine treatment regimen for *S. stercoralis* and *Taenia* spp. since the exposure of the parasites to sub-curative doses exacerbates the risk of resistance development. Therefore, pharmacovigilance needs to also cover non-target parasites to assure timely detection of emerging resistance.

## Supporting Information

Checklist S1 CONSORT Checklist
(0.13 MB PDF)

Alternative Language Abstract S1 Translation of the Abstract into Chinese by Shu-Hua Xiao
(0.05 MB PDF)

Alternative Language Abstract S2 Translation of the Abstract into German by Peter Steinmann
(0.04 MB PDF)

Protocol S1 This is the study protocol of the research reported as submitted to the Ethics Committee of the Unviversity and State of Basel (EKBB) on 3 May 2007.
(0.37 MB DOC)
