# Supplementary material for: Tribendimidine and Albendazole for Treating Soil-Transmitted Helminths, Strongyloides stercoralis and Taenia spp.: Open-Label Randomized Trial
Source: PLoS Negl Trop Dis. 2008 Oct 15;2(10):e322. doi: 10.1371/journal.pntd.0000322 (PMC2561005; doi:10.1371/journal.pntd.0000322)
Supplement: Checklist S1 — CONSORT Checklist (0.13 MB PDF) [file pntd.0000322.s001.pdf]

CONSORT Statement 2001 - Checklist

Items to include when reporting a randomized trial

PAPER SECTION And topic	Item	Descriptor	Reported on Page #
TITLE & ABSTRACT	1	How participants were allocated to interventions (*e.g.*, "random allocation", "randomized", or "randomly assigned").	1, 3
INTRODUCTION Background	2	Scientific background and explanation of rationale.	5, 6
METHODS Participants	3	Eligibility criteria for participants and the settings and locations where the data were collected.	7, 9, 10
Interventions	4	Precise details of the interventions intended for each group and how and when they were actually administered.	7, 8, 9
Objectives	5	Specific objectives and hypotheses.	6, 7
Outcomes	6	Clearly defined primary and secondary outcome measures and, when applicable, any methods used to enhance the quality of measurements (*e.g.*, multiple observations, training of assessors).	7, 8, 10
Sample size	7	How sample size was determined and, when applicable, explanation of any interim analyses and stopping rules.	7
Randomization -- Sequence generation	8	Method used to generate the random allocation sequence, including details of any restrictions (*e.g.*, blocking, stratification)	8
Randomization -- Allocation concealment	9	Method used to implement the random allocation sequence (*e.g.*, numbered containers or central telephone), clarifying whether the sequence was concealed until interventions were assigned.	8, 9
Randomization -- Implementation	10	Who generated the allocation sequence, who enrolled participants, and who assigned participants to their groups.	8, 9
Blinding (masking)	11	Whether or not participants, those administering the interventions, and those assessing the outcomes were blinded to group assignment. If done, how the success of blinding was evaluated.	9
Statistical methods	12	Statistical methods used to compare groups for primary outcome(s); Methods for additional analyses, such as subgroup analyses and adjusted analyses.	10
RESULTS Participant flow	13	Flow of participants through each stage (a diagram is strongly recommended). Specifically, for each group report the numbers of participants randomly assigned, receiving intended treatment, completing the study protocol, and analyzed for the primary outcome. Describe protocol deviations from study as planned, together with reasons.	11, 25
Recruitment	14	Dates defining the periods of recruitment and follow-up.	7
Baseline data	15	Baseline demographic and clinical characteristics of each group.	11, 26
Numbers analyzed	16	Number of participants (denominator) in each group included in each analysis and whether the analysis was by "intention-to-treat". State the results in absolute numbers when feasible (*e.g.*, 10/20, not 50%).	11, 28
Outcomes and estimation	17	For each primary and secondary outcome, a summary of results for each group, and the estimated effect size and its precision (*e.g.*, 95% confidence interval).	12, 13
Ancillary analyses	18	Address multiplicity by reporting any other analyses performed, including subgroup analyses and adjusted analyses, indicating those pre-specified and those exploratory.	12
Adverse events	19	All important adverse events or side effects in each intervention group.	13, 14
DISCUSSION Interpretation	20	Interpretation of the results, taking into account study hypotheses, sources of potential bias or imprecision and the dangers associated with multiplicity of analyses and outcomes.	14-18
Generalizability	21	Generalizability (external validity) of the trial findings.	15, 17
Overall evidence	22	General interpretation of the results in the context of current evidence.	17
