# Supplementary material for: Tribendimidine and Albendazole for Treating Soil-Transmitted Helminths, Strongyloides stercoralis and Taenia spp.: Open-Label Randomized Trial
Source: PLoS Negl Trop Dis. 2008 Oct 15;2(10):e322. doi: 10.1371/journal.pntd.0000322 (PMC2561005; doi:10.1371/journal.pntd.0000322)
Supplement: Alternative Language Abstract S1 — Translation of the Abstract into Chinese by Shu-Hua Xiao (0.05 MB PDF) [file pntd.0000322.s002.pdf]

三苯双脒和阿苯达唑治疗土源性传播的蠕虫、粪类圆线虫（*Strongyloides stercoralis*）和带绦虫〔*Taenia* spp.〕感染：开放随机对比临床试验

摘要

背景：三苯双脒为一广谱抗蠕虫药物，并于 2004 年为中国食品药品管理局所批准。三苯双脒对土源性传播的蠕虫（蛔虫、钩虫和鞭虫）感染的疗效业已确定.新的实验研究表明三苯双脒具有抗绦虫和大鼠粪类圆线虫(*Strongyloides ratti*)的作用.

方法学/主要的发现: 在中华人民共和国云南省的一个乡村,用开放随机对比的临床试验评价口服单剂阿苯达唑和三苯双脒 (5-14 岁儿童服 200 mg,≥15 岁服 400 mg)对土源性传播的蠕虫、人的粪类圆线虫(*Strongyloides stercoralis*)和带绦虫(*Taenia* spp.)的疗效。资料分析是在符合方案集(PP)的基础上进行，本临床试验已在 controlled-trials.com 登记(登记号为 ISRCTN01779485)。阿苯达唑和三苯双脒的单剂量对人蛔虫有高效，对钩虫有中等疗效，而对鞭虫的疗效低。 在接受三苯双脒治疗的 57 例中，粪类圆线虫的感染率由 19.3％降至 8.8％（观察的治愈率为 54.5％，*P*＝0.107）,而带绦虫的则由 26.3%降至 8.8%(观察的治愈率为 66.7％,*P*=0.014)。用阿苯达唑治疗的 66 例亦有相似的粪类圆线虫和带绦虫感染率的降低。在疗效考核中发现“新感染者”的最大可能性是由于所用诊断方法不够敏感，未能于治前将其检出。在药物治疗带绦虫感染净治愈率间的差异，三苯双脒具有高度显著性（*P*＝0.001）。未观察到 2 种药物有明显的不良反应。

结论/意义：我们的观察结果表明，三苯双脒单剂口服可用于有广泛的多肠道寄生虫感染的地区，其对蛔虫和钩虫感染的疗效已被证实。用三苯双脒治疗所获得的对粪类圆线虫和带绦虫有良好的效果值得进一步研究。下一步应对三苯双脒多剂量的治疗方案作出评价。

关键词 三苯双脒；阿苯达唑；土源性传播的蠕虫；粪类圆线虫；带绦虫；中华人民共和国
