# Supplementary material for: Tribendimidine and Albendazole for Treating Soil-Transmitted Helminths, Strongyloides stercoralis and Taenia spp.: Open-Label Randomized Trial
Source: PLoS Negl Trop Dis. 2008 Oct 15;2(10):e322. doi: 10.1371/journal.pntd.0000322 (PMC2561005; doi:10.1371/journal.pntd.0000322)
Supplement: Alternative Language Abstract S2 — Translation of the Abstract into German by Peter Steinmann (0.04 MB PDF) [file pntd.0000322.s003.pdf]

Behandlung von durch Bodenkontakt übertragenen Wurmerkrankungen, *Strongyloides stercoralis* und *Taenia* spp. mit Tribendimidine und Albendazole: Resultate einer randomisierten Studie

Zusammenfassung

Hintergrund: Tribendimidine ist ein Medikament gegen Wurminfektionen. Es hat ein breites Wirkungsspektrum und wurde im Jahr 2004 von den chinesischen Arzneimittelbehörden zugelassen. Seine Wirksamkeit gegen die durch Bodenkontakt übertragenen Würmer *Ascaris lumbricoides*, Hakenwürmer und *Trichuris trichiura* ist gut dokumentiert. Neue Laborstudien deuten darauf hin, dass Tribendimidine auch gegen Bandwürmer und *Strongyloides ratti* wirken könnte.

Methoden/wichtigste Ergebnisse: In einer randomisierten Studie wurden die Verträglichkeit und Wirksamkeit von Albendazole und Tribendimidine in Einmaldosen untersucht. Beide Medikamente wurden in einer Dosierung von 200 mg (5 bis 14-jährige Kinder) oder 400 mg (≥15-jährige Teilnehmer) abgegeben, und deren Wirksamkeit gegen durch Bodenkontakt übertragene Würmer, *S. stercoralis* und *Taenia* spp. untersucht. Die Studie wurde in einem Dorf in der Provinz Yunnan in der Volksrepublik China durchgeführt. Die Studie ist bei „controlled-trials.com" unter der Nummer ISRCTN01779485 registriert. Einmaldosen Albendazole und Tribendimidine waren hoch wirksam gegen *A. lumbricoides* und zeigten eine moderate Aktivität gegen Hakenwürmer. Gegen *T. trichiura* resultierte keine oder lediglich eine schwache Wirksamkeit. Die Prävalenz von *S. stercoralis* unter den 57 mit Tribendimidine behandelten Individuen sank von 19.3% auf 8.8% (beobachtete Heilungsrate: 54.5%, p=0.107) während diejenige von *Taenia* spp. von 26.3% auf 8.8% reduziert wurde (beobachtete Heilungsrate: 66.7%, p=0.014). Eine vergleichbare Prävalenzreduktion wurde

bei den 66 mit Albendazole behandelten Studienteilnehmern beobachtet. Während der Evaluation der Behandlung wurden aber auch „neue" Infektionen diagnostiziert. Vermutlich waren diese vor der Behandlung aufgrund der mangelhaften Sensitivität der verfügbaren diagnostischen Mittel nicht erfasst worden. Deren Berücksichtigung resultierte in einem hoch signifikanten Unterschied zwischen den Medikamenten-spezifischen Heilungsraten im Falle von *Taenia* spp. Tribendimidine war weit wirksamer als Albendazole (p=0.001). Es wurden keinerlei negativen Nebenwirkungen der Medikation beobachtet.

Schlussfolgerungen/Bedeutung: Die vorgelegten Resultate deuten darauf hin, dass Einzeldosen Tribendimidine in Gebieten mit einer hohen Multiparasitismus-Prävalenz eingesetzt werden können. Die Wirksamkeit des Medikamentes gegen *A. lumbricoides* und Hakenwürmer wurde bestätigt. Die viel versprechenden Resultate der Behandlung von *S. stercoralis* und *Taenia* mit Tribendimidine rechtfertigen weitere Studien. Als nächstes sollten Behandlungsschemata mit mehrfacher Dosierung untersucht werden.

Übersetzung: Peter Steinmann
