# Supplementary material for: Tribendimidine and Albendazole for Treating Soil-Transmitted Helminths, Strongyloides stercoralis and Taenia spp.: Open-Label Randomized Trial
Source: PLoS Negl Trop Dis. 2008 Oct 15;2(10):e322. doi: 10.1371/journal.pntd.0000322 (PMC2561005; doi:10.1371/journal.pntd.0000322)

**Research protocol**

Swiss Tropical Institute (STI; Basel, Switzerland) and National Institute of Parasitic Diseases (IPD; Shanghai, China)

**Title**

**Epidemiology and treatment of soil-transmitted helminthiasis, with particular consideration to strongyloidiasis, in Yunnan province, China**

**Appendices**

**Appendix 1:** Informed consent sheet

**Appendix 2:** Registration forms (participation, laboratory)

**Appendix 3:** Summary and grading of adverse side effects

**Investigators**

**Prof. Jürg Utzinger (Epidemiologist; Co-investigator)**

Department of Public Health and Epidemiology

Swiss Tropical Institute

Socinstrasse 57

CH- 4002 Basel, Switzerland

Tel.: +41 61 284-8129

Fax: +41 61 284-8105

E-mail: juerg.utzinger@unibas.ch

**Peter Steinmann (Epidemiologist; Co-investigator)**

Department of Public Health and Epidemiology

Swiss Tropical Institute

Socinstrasse 57

CH- 4002 Basel, Switzerland

Tel.: +41 61 284-8226

Fax: +41 61 284-8105

E-mail: peter.steinmann@unibas.ch

**Prof. Xiao-Nong Zhou (Epidemiologist and Deputy Director; Co-investigator)**

National Institute of Parasitic Diseases

Chinese Center for Disease Control and Prevention

207 Rui Jin Er Road

Shanghai 200025, People’s Republic of China

Tel.: +86 21 6473-8058

Fax: +86 21 6433-2670

Email: ipdzhouxn@sh163.net

**Prof. Shu-Hua Xiao (Pharmacologist and Experimental Parasitologist; Co-investigator)**

National Institute of Parasitic Diseases

Chinese Center for Disease Control and Prevention

207 Rui Jin Er Road

Shanghai 200025, People’s Republic of China

Tel.: +86 21 6435-6308

Fax: +86 21 6433-2670

Email: shxiao1@yahoo.com

**Prof. Zun-Wei Du (Parasitologist, Co-investigator)**

Yunnan Institute of Parasitic Diseases

Chinese Center for Disease Control and Prevention

6 Xiyuan Road

Simao (Pu’er) 665000; People’s Republic of China

Tel.: +86 87 9212-2153

Fax: +86 87 9212-2153

Email: dzw5509@163.com

**1. Background**

Soil-transmitted helminthiasis, caused by infections with intestinal nematodes, remains among the most prevalent and debilitating parasitic diseases in the world (Horton 2003; Utzinger and Keiser 2004; Bethony et al. 2006). More than 2 billion people are infected, and there is growing evidence about the negative impact of soil-transmitted helminth infections on the course and outcome of pregnancy (i.e. anaemia), children’s physical and cognitive development, educational attainment and worker productivity in later life (WHO 2002; Miguel and Kremer 2004; Utzinger and Keiser 2004; Bethony et al. 2006; Lammie et al. 2006). Recent estimates suggest that the global burden due to soil-transmitted helminthiasis might by as high as 39 million disability-adjusted life years (DALYs), thus approaching or even surpassing the burden of malaria (Hotez et al. 2006). The major soil-transmitted helminth species are *Ascaris lumbricoides*, *Trichuris trichiura* and the hookworms (*Necator americanus* and *Ancylostoma duodenale*). They show the widest geographical distribution and current estimates are that between 807 and 1221 million people are infected with *A. lumbricoides*, 604-795 million people with *T. trichiura*, and 576-740 million people with the hookworms (Bethony et al. 2006).

There is growing recognition of the public-health significance of yet another soil-transmitted helminth, namely *Strongyloides stercoralis* (Keiser and Nutman 2004). An estimated 30-100 million people are infected (Bethony et al. 2006). However, no data is currently available regarding the global burden and geographical distribution of strongyloidiasis. An important underlying reason is that one of the most widely used diagnostic approaches for the evaluation of intestinal helminth infections – i.e. the Kato-Katz technique (Katz et al. 1972) – is unsuitable for detection of *S. stercoralis*, and microscopic examination of direct faecal smears only has a low sensitivity (Sato et al. 1995). The available methods with a high sensitivity [i.e. Koga agar plate (Koga et al. 1991) and Baermann technique (Garcia 2001)] are time-consuming and somewhat cumbersome to perform.

High prevalences of *A. lumbricoides*, *T. trichiura* and hookworms have been reported from sub-Saharan Africa and Southeast Asia, including China (de Silva et al. 2003; Bethony et al. 2006). According to the Chinese Ministry of Health (MoH) the prevalence of soil-transmitted helminth infections in Yunnan province was 21.7% during the second nationwide parasitological survey completed in 2004 (MoH 2005). Additional helminth infections are known to occur in this area, i.e. *Enterobius vermicularis*, *Taenia solium* and *T. saginata*, and the presence of various food-borne trematodes is strongly suspected. There is a lack of data for *S. stercoralis*.

In June 2006, we carried out a cross-sectional study in a small village in the southern part of the Yunnan province. Parasitological screening of 180 randomly selected individuals revealed that soil-transmitted helminth infections were pervasive; we found a point prevalence of *A. lumbricoides*, *T. trichiura* and hookworms of 93.9%, 88.9% and 87.8%, respectively. In addition, we found 21 cases positive for *S. stercoralis*, accounting for a prevalence of 11.7% (Steinmann et al. 2007). Our results implied that multiparasitism was the norm rather than the exception, confirming recent reports from Africa (Raso et al. 2004) and South America (Brooker et al. 2006).

The functional significance of multiple helminth infections on clinical outcomes (e.g. anaemia) are increasingly appreciated (Ezeamama et al. 2005) and opportunities offered for integrated control approaches have been discussed (Hotez et al. 2006; Lammie et al. 2006; Utzinger and de Savigny 2006).

Current efforts for the control of soil-transmitted helminth infections rely on chemotherapy (Lammie et al. 2006; Utzinger and de Savigny 2006). Albendazole and mebendazole are routinely used in chemotherapy campaigns targeting millions of children and adults each year. These drugs have a good safety profile and the low frequency and generally mild nature of adverse side effects even allows their use by non-medical personnel, such as school teachers in school-based mass treatment campaigns (WHO 2002). Albendazole shows high efficacies against *A. lumbricoides* and the hookworms and a moderate efficacy against *T. trichiura* (Utzinger and Keiser 2004). Unfortunately, albendazole only has a low-to-moderate efficacy against *S. stercoralis*. The standard treatment for the latter parasite is ivermectin. This drug, on the other hand, exhibits only low activity against other soil-transmitted helminths (Utzinger and Keiser 2004).

Tribendimidine is a broad-spectrum anthelminthic drug developed by Chinese scientists in the mid-1980s (Ren et al. 1987). Tribendimidine is structurally related to amidantel, which has been developed by Bayer in the late 1970s and has been effectively used against various helminths (nematodes and trematodes) in laboratory studies and clinical trials (Thomas 1979; Wollweber et al. 1979; Rim et al. 1980). Tribendimidine is licensed by the Chinese Food and Drug Administration for the treatment of human infections with *A. lumbricoides*, *T. trichiura* and the hookworms. Its safety record compares well with that of albendazole (Xiao et al. 2005), which is further underscored by recent data obtained from phase IV trials. Clinical studies revealed that tribendimidine shows promissing activity against *Taenia* spp. and *E. vermicularis*. Unpublished studies further indicate high activities of tribendimidine against *Strongyloides venezuelensis, Hymenolepis nana* and *H. diminuta* in mice and rats. The activity of tribendimidine against *S. stercoralis* has not yet been assessed.

The World Health Organization (WHO) recommends that anthelminthic drugs are administered to entire populations in case the prevalence of soil-transmitted helminths (i.e. *A. lumbricoides*, *T. trichiura* and hookworm infection) exceeds 50% (WHO 2002). Ancillary benefits are likely to occur among individuals who are concurrently infected with other parasites (e.g. *S. stercoralis*) in case the anthelminthic drug has some efficacy against these parasites. In settings where the prevalence of soil-transmitted helminthiasis is high, small studies to comparatively assess the efficacy of different anthelmintic drugs are useful and can guide subsequent policy recommendations. This issue has been documented for Vietnam, where mebendazole is the current drug of choice in the national helminth control programme, despite significantly lower efficacy when compared to albendazole (Flohr et al. 2007).

We proposed to assess the safety and efficacy of albendazole versus tribendimidine against soil-transmitted helminthiasis, including *S. stercoralis*. Our proposal is further motivated by the following considerations. First, it is plausible that tribendimidine will be used in mass treatment campaigns in China, in addition to the currently used albendazole. Since tribendimidien and albendazole belong to different chemical classes (Wollweber et al. 1979; Utzinger and Keiser 2004), alternating between the two drugs would reduce the chance of resistance development (Geerts and Gryseels 2000). Second, by default, mass treatment campaigns are not preceded by individual diagnosis of parasitic infections, and hence there is an opportunity to assess the potential ancillary benefits on other parasitic infections. Third, we lack detailed knowledge on whether multiple species parasitic infections have an effect on treatment outcomes (i.e. altered safety or efficacy profiles). Fourth, in case tribendimidine results in a higher efficacy against *S. stercoralis* than albendazole, the use of tribendimidine would offer an elegant way to cover an additional parasite, which is neglected, but nonetheless of considerable public health significance. It is important to note that ivermectin is currently not available for human use in China.

**2. Detailed research plan**

**2.1. Study aims**

(1) To further our understanding of the local epidemiology of soil-transmitted helminthiasis (including strongyloidiasis) in selected villages in Yunnan province of China.

(2) To comparatively assess the sensitivity and specificity of different diagnostic approaches for soil-transmitted helminth infections, with particular consideration to *S. stercoralis*.

(3) To assess the safety and efficacy of oral albendazole versus oral tribendimidine (both drugs will be administered in a single dose of 200 mg for children aged 5-14 years, and 400 mg for individuals aged ≥15 years) against different soil-transmitted helminth infections.

**2.2. Experimental design**

*2.2.1. Study area and population surveyed*

The study will be carried out in Nan Weng village in Menghai county, Xishuangbanna prefecture, Yunnan province, China. The village is inhabited by approximately 350 individuals of Bulang nationality, belonging to 80 families. All family members aged 2 years and above will be invited to participate in the initial parasitological screening. Participating (≥1 stool sample submitted) individuals aged ≥5 years will be enrolled in the comparative treatment efficacy study, irrespective of the parasitological diagnosis.

Based on our preliminary work in the same area we anticipate that 80-90% of the eligible individuals who are present in the village at the time of the survey will submit at least 1 stool sample and we anticipate that point prevalence with the three main soil-transmitted helminths will exceed 80%. Moreover, we anticipate that 10-15% of the individuals will harbour *S. stercoralis*.

*2.2.2. Patient inclusion criteria*

(1) Age ≥5 years, both sexes.

(2) Submission of ≥1 stool sample for the baseline parasitological survey.

(3) For females, not pregnant, as verbally assessed by medical personnel on the day of treatment.

(4) Absence of major systemic illnesses, as assessed by medical personnel on the day of treatment.

(5) Written informed consent by the head of the household on behalf of the whole family.

*2.2.3. Patient exclusion criteria*

(1) Presence of any abnormal medical condition, as judged by the medical personnel.

(2) No stool sample submitted for baseline parasitological survey.

(3) Enrolled in any other clinical investigation during the study.

(4) For females: pregnancy

Standard anthelminthic treatment according to the applicable Chinese treatment guidelines will be offered to all residents of Nan Weng village aged 2 years and above who are not included in the comparative treatment efficacy study.

*2.2.4. Baseline survey*

Demographic, economic and health-related behavioural data of the village population will be collected by means of pre-tested individual and family questionnaires. All residents aged ≥2 years will be invited to submit 3 stool samples over a 6-day period. The stool samples will be collected daily and continuously processed by the following diagnostic techniques.

(1) Kato-Katz (diagnosis of *A. lumbricoides*, *T. trichiura*, hookworms, *Taenia* spp., other helminths): 1 slide/sample.

(2) Baermann (diagnosis of *S. stercoralis*): 1 test/sample.

(3) Koga agar plate (diagnosis of *S. stercoralis* and hookworms): 1 plate/sample.

(4) Sodium acetate-acetic acid-formaline (SAF) conservation (followed by ether-sedimentation technique, to be done after conclusion of the field study; diagnosis of helminths and intestinal protozoa): 1 sample/individual

*2.2.5. Treatment*

Study participants (according to above-specified inclusion criteria) will be randomly assigned to either albendazole (group A) or tribendimidine (group B).

In group A, participants aged 5-14 years will receive a single 200 mg oral dose of albendazole; older participants will be treated with a single 400 mg oral dose of albendazole. In group B, participants aged 5-14 years will receive a single 200 mg oral dose of tribendimidine; older participants will be treated with a single 400 mg oral dose of tribendimidine. The drugs will be distributed to the study participants after dinner time (ca. 18:00 hours) by local health professionals, and ingestion confirmed.

*2.2.6. Acute adverse effect monitoring*

The treating health professionals and a medical doctor will be present in the village for the next 3 hours following drug administration and respond to requests for medical assistance in case of acute adverse events (AEs). All study participants will be invited to report AEs 24 hours post-treatment. In case of AEs, appropriate medical action will be taken, e.g. administration of drugs for relief.

AEs will be graded, and their onset and duration recorded (see Appendix 3). The intensity of AEs will be judged by the medial doctor as followed:

*Mild:* present but not requiring any interference

*Moderate:* (1) present and requiring medication for symptomatic relief as requested by the affected people or 2) present and interfering with normal daily activities.

*Severe:* present and required medical intervention beyond symptomatic relief.

.

*2.2.7. Endpoint parasitological stool survey*

2-4 weeks after the treatment, all study participants will be invited to submit another 3 stool samples over a 6-day period. The stool samples will be collected daily and continuously processed by the same diagnostic techniques as detailed above.

Participants without eggs or larvae in their stools as assessed by the respective methods will be considered parasitologically cured. Egg reduction rates for the different parasites will be calculated based on the arithmetic mean egg count of the 3 Kato-Katz slides prepared before and after treatment. Cure rates will be calculated on a per-species basis and not take into account the influence of the treatment on any co-infections.

*2.2.8. Concomitant therapy*

No restriction except antiparasitic treatment (albendazole, mebendazole, levamisole, pyrantel pamoate or any other anthelminthic drug). Participants are asked about antiparasitic treatment over the preceding month upon distribution of the study drugs.

**3. References**

Bethony, J., Brooker, S., Albonico, M., Geiger, S.M., Loukas, A., Diemert, D., Hotez, P.J. (2006). Soil-transmitted helminth infections: ascariasis, trichuriasis, and hookworm. Lancet **367**: 1521-32.

Brooker, S., Alexander, N., Geiger, S., Moyeed, R.A., Stander, J., Fleming, F., Hotez, P.J., Correa-Oliveira, R., Bethony, J. (2006). Contrasting patterns in the small-scale heterogeneity of human helminth infections in urban and rural environments in Brazil. Int J Parasitol **36**:1143-51

de Silva, N.R., Brooker, S., Hotez, P.J., Montresor, A., Engels, D., Savioli, L. (2003). Soil-transmitted helminth infections: updating the global picture. Trends Parasitol **19:** 547-51.

Ezeamama, A.E., Friedman, J.F., Olveda, R.M., Acosta, L.P., Kurtis, J.D., Mor, V., McGarvey, S.T. (2005). Functional significance of low-intensity polyparasite helminth infections in anemia. J Infect Dis **192:** 2160-70.

Flohr, C., Tuyen, L.N., Lewis, S., Minh, T.T., Campbell, J., Britton, J., Williams, H., Hien, T.T., Farrar, J., Quinnell, R.J. (2007). Low efficacy of mebendazole against hookworm in Vietnam: two randomized controlled trials. Am J Trop Med Hyg 76: 732-6.

Garcia, L.S. (2001). Diagnostic medical parasitology. Washington, D.C., ASM Press.

Geerts, S., Gryseels, B. (2000). Drug resistance in human helminths: current situation and lessons from livestock. Clin Microbiol Rev **13**: 207-22.

Horton, J. (2003). Human gastrointestinal helminth infections: are they now neglected diseases? Trends Parasitol **19:** 527-31.

Hotez, P.J., Molyneux, D.H., Fenwick, A., Ottesen, E., Ehrlich Sachs, S., Sachs, J.D. (2006). Incorporating a rapid-impact package for neglected tropical diseases with programs for HIV/AIDS, tuberculosis, and malaria. PLoS Med **3**: e102.

Katz, N., Chaves, A., Pellegrino, J. (1972). A simple device for quantitative stool thick-smear technique in schistosomiasis mansoni. Rev Inst Med Trop Sao Paulo **14:** 397-400.

Keiser, P.B., Nutman, T.B. (2004). *Strongyloides stercoralis* in the immunocompromised population. Clin Microbiol Rev **17:** 208-17.

Koga, K., Kasuya, S., Khamboonruang, C., Sukhavat, K., Ieda, M., Takatsuka, N., Kita, K., Ohtomo, H. (1991). A modified agar plate method for detection of *Strongyloides stercoralis*. Am J Trop Med Hyg **45:** 518-21.

Lammie, P.J., Fenwick, A., Utzinger, J. (2006). A blueprint for success: integration of neglected tropical disease control programmes. Trends Parasitol **22**: 313-21.

Miguel, E., Kremer, M. (2004). Worms: identifying impacts on education and health in the presence of treatment externalities. Econometrica **72:** 159-217.

MoH (2005). Report on the national survey of current situation of major human parasitic diseases in China. National Institute of Parasitic Diseases, China CDC, Shanghai**:** 1-33.

Raso, G., Luginbühl, A., Adjoua, C.A., Tian-Bi, N.T., Silué, K.D., Matthys, B., Vounatsou, P., Wang, Y., Dumas, M.E., Holmes, E., Singer, B.H., Tanner, M., N'Goran, E.K., Utzinger, J. (2004). Multiple parasite infections and their relationship to self-reported morbidity in a community of rural Côte d'Ivoire. Int J Epidemiol **33:** 1092-102.

Ren, H.N., Cheng, B.Z., Zhuang, Z.N. (1987). Experimental therapeutic efficacy of a new anti-hookworm drug – tribendimidin. Chin J Parasitol Parasit Dis **5:** 262-4.

Rim, H.J., Joo, K.H., Kim, Y.Y., Lee, J.S., Song, S.D. (1980). Anthelmintic effect of amidantel (Bay d 8815) against *Ancylostoma duodenale* infection. Korean J Parasitol **18:** 24-36.

Sato, Y., Kobayashi, J., Toma, H., Shiroma, Y. (1995). Efficacy of stool examination for detection of *Strongyloides* infection. Am J Trop Med Hyg **53:** 248-50.

Steinmann, P., Zhou, X.N., Du, Z.W., Jiang, J.Y., Wang, L.B., Wang, X.Z., Li, L.H., Marti, H., Utzinger, J. (2007). Epidemiology of *Strongyloides stercoralis* in Yunnan province, China. PLoS Neglect Trop Dis (submitted for publication).

Thomas, H. (1979). The efficacy of amidantel, a new anthelmintic, on hookworms and ascarids in dogs. Tropenmed Parasitol **30:** 404-8.

Utzinger, J., de Savigny, D. (2006). "Control of neglected tropical diseases: integrated chemotherapy and beyond." PLoS Med **3**: e112.

Utzinger, J., Keiser, J. (2004). Schistosomiasis and soil-transmitted helminthiasis: common drugs for treatment and control. Expert Opin Pharmacother **5**: 263-85.

WHO (2002). Prevention and control of schistosomiasis and soil-transmitted helminthiasis. World Health Organization, Geneva**:** WHO Tech Rep Ser No. 912: 1-57.

Wollweber, H., Niemers, E., Flucke, W., Andrews, P., Schulz, H.P., Thomas, H. (1979). Amidantel, a potent anthelminthic from a new chemical class. Drug Res **29:** 31-2.

Xiao, S.H., Wu, H.M., Tanner, M., Utzinger, J., Wang, C. (2005). Tribendimidine: a promising, safe and broad-spectrum anthelminthic agend from China. Acta Trop **94**: 1-14.

**Appendix 1: Informed consent** (translated to Chinese)

**Study participant information and consent sheet**

COMPARISON OF THE DIAGNOSTIC PERFORMANCE
OF DIFFERENT METHODS FOR THE DETECTION OF INTESTINAL PARASITES
WIT A SPECIAL FOCUS ON *STRONGYLOIDES STERCORALIS*

EVALUATION OF THE TREATMENT EFFICACY OF 2 COMMONLY USED ANTI-PARASITIC DRUGS

**This study is jointly undertaken by researchers from the following institutions:**

- Yunnan Institute for Parasitic Diseases (YIPD), China CDC, Simao, People’s Republic of China

- National Institute of Parasitic Diseases (IPD), China CDC, Shanghai, People’s Republic of China

- Swiss Tropical Institute (STI), Basel, Switzerland

**Principal investigators:** The following persons are directly responsible for the design and the implementation of the study: Prof. Dr. Du Zun-Wei (YIPD), Prof. Dr. Zhou Xiao-Nong (IPD), Prof. Dr. Jürg Utzinger (STI) and Peter Steinmann, PhD-Student (STI).

**Aim of the study:** Helminth infections (small parasitic worms such as hookworms, roundworms, tapeworms, etc.) and intestinal protozoa live in the intestine and are causing a wide range of diseases and conditions ranging from mild and unnoticed to severe and debilitating. Among the main symptoms are intestinal problems, general weakness and fatigue. There is no single diagnostic method capable of reliably detecting all intestinal parasites and *Strongyloides stercoralis* is missed by most traditional methods. It is also known that the excretion of parasites is not uniform, thus infections can be missed if only one stool sample is analyzed. We want to compare the performance of different diagnostic methods and to assess how many additional infections are diagnosed if 3 instead of 1 stool sample are examined. Safe drugs can be used to treat helminth infections. We want to compare the efficacy of two drugs which are licensed for human use by the China State Food and Drug Administration. These two drugs are albendazole and tribendimidine.

For this study we recruit the members aged 2 years and above of all families living in your village.

**Procedure:** We will ask you and every member of your family aged ≥2 years who is not pregnant to provide us your morning stool over several days (total of 3 stool samples). In addition, we will ask questions about your living conditions, personal health and habits. We will then check the stools for the eggs of the different worms or their larvae.

Every participant aged 5 years and above will be treated free of charge with one of the two drugs but you do not know which drug you get. We will ask you 3 and 24 hours after the treatment if you felt any side effects of the treatment. 2-4 weeks after the treatment, we will again ask you to submit 3 stool samples and analyze them using the same methods as before. If you are still infected, you will be re-treated with albendazole. Children aged 2-4 years will be treated with albendazole according to the Chinese national standard.

**Confidentiality:** The data collected may be published but no names will appear in any publications. No personal data and diagnoses will be made available in a way that allows the identification of real persons to other people, authorities or companies than those directly involved in the study.

**Potential risk:** There is no risk associated with the participation in this study. The use of both drugs should be safe even in pregnant women. Still, pregnant members of your family should not participate.

**Participation/withdrawal:** Participation in this study is entirely voluntary and you or any member of your household is free to withdraw from the study at any moment without further obligations.

**Costs/compensation:** Participation in this study will not result in any costs to you. The treatment of diagnosed worms is free of charge.

**Contact person for further questions and complaints:** Any questions or complaints can be directed at the fieldworkers who will try to answer them or transfer them to one of the principal investigators. You may also wish to contact directly Prof. Dr. Zhou Xiao-Nong anytime, office: +86 21 64738058, mobile: 13916230620.

**I** (please print) **_____________have read and understood this text and all my questions have been answered. I am aware of my rights and duties and I freely accept to participate in this study with my family.**

**Date and signature participant (head of household):**

**Date and signature fieldworker:**

Appendix 2: Overview family and participation

| **Village:__________________** | |  |  |  |  |  |  |  |  |  |  |
| --- | --- | --- | --- | --- | --- | --- | --- | --- | --- | --- | --- |
| **Family name:______________________** | | | **Family ID:__________** | |  |  |  |  |  |  |  |
|  |  |  |  |  |  |  |  |  |  |  |  |
| **Family members** | |  |  |  |  |  |  |  |  |  |  |
|  |  |  |  |  |  |  |  |  |  |  |  |
|  | **Name** | **ID** | **Year of birth** | **Not partici-pating** | **Sample A** | **Sample B** | **Sample C** | **Treatment** | **Sample D** | **Sample E** | **Sample F** |
| **1** |  |  |  |  |  |  |  |  |  |  |  |
| **2** |  |  |  |  |  |  |  |  |  |  |  |
| **3** |  |  |  |  |  |  |  |  |  |  |  |
| **4** |  |  |  |  |  |  |  |  |  |  |  |
| **5** |  |  |  |  |  |  |  |  |  |  |  |
| **6** |  |  |  |  |  |  |  |  |  |  |  |
| **7** |  |  |  |  |  |  |  |  |  |  |  |
| **8** |  |  |  |  |  |  |  |  |  |  |  |
| **9** |  |  |  |  |  |  |  |  |  |  |  |
| **10** |  |  |  |  |  |  |  |  |  |  |  |

| **Kato-Katz** | |  |  |  |  |  |  |  |
| --- | --- | --- | --- | --- | --- | --- | --- | --- |
|  |  |  |  |  |  |  |  |  |
| Instructions: Count all eggs on the slide and note the number in the appropriate field | | | | | |  |  |  |
|  |  |  |  |  |  |  |  |  |
| **Technician:** | |  |  |  |  | **Date:** | |  |
|  |  |  |  |  |  |  |  |  |
| **ID number** | ***Ascaris  lumbricoides*** | **Hookworm** | ***Trichuris trichiura*** | ***Taenia* spp.** | **Other** | | **Other** | |
|  |  |  |  |  | **Name** | **Egg count** | **Name** | **Egg count** |
|  |  |  |  |  |  |  |  |  |
|  |  |  |  |  |  |  |  |  |
|  |  |  |  |  |  |  |  |  |
|  |  |  |  |  |  |  |  |  |
|  |  |  |  |  |  |  |  |  |
|  |  |  |  |  |  |  |  |  |
|  |  |  |  |  |  |  |  |  |
|  |  |  |  |  |  |  |  |  |
|  |  |  |  |  |  |  |  |  |
|  |  |  |  |  |  |  |  |  |
|  |  |  |  |  |  |  |  |  |
|  |  |  |  |  |  |  |  |  |
|  |  |  |  |  |  |  |  |  |
|  |  |  |  |  |  |  |  |  |
|  |  |  |  |  |  |  |  |  |
|  |  |  |  |  |  |  |  |  |
|  |  |  |  |  |  |  |  |  |

| **Koga culture** |  |  |  |  |  |  |
| --- | --- | --- | --- | --- | --- | --- |
|  |  |  |  |  |  |  |
| Instructions: check plate for larvae and their tracks, count larvae in sediment and differentiate | | | | | | |
| In the last two columns, give the number of identified larvae of both species | | | | | |  |
|  |  |  |  |  |  |  |
| **Technician:** | | | |  | **Date:** | |
|  |  |  |  |  |  |  |
| **ID number** | **Tracks** | | **No of larvae (plate)** | | **No of larvae (sediment)** | |
|  | Yes | No | ***Strongyloides*** | **Hookworm** | ***Strongyloides*** | **Hookworm** |
|  |  |  |  |  |  |  |
|  |  |  |  |  |  |  |
|  |  |  |  |  |  |  |
|  |  |  |  |  |  |  |
|  |  |  |  |  |  |  |
|  |  |  |  |  |  |  |
|  |  |  |  |  |  |  |
|  |  |  |  |  |  |  |
|  |  |  |  |  |  |  |
|  |  |  |  |  |  |  |
|  |  |  |  |  |  |  |
|  |  |  |  |  |  |  |
|  |  |  |  |  |  |  |
|  |  |  |  |  |  |  |
|  |  |  |  |  |  |  |
|  |  |  |  |  |  |  |
|  |  |  |  |  |  |  |
|  |  |  |  |  |  |  |
|  |  |  |  |  |  |  |
|  |  |  |  |  |  |  |
|  |  |  |  |  |  |  |
|  |  |  |  |  |  |  |
|  |  |  |  |  |  |  |
|  |  |  |  |  |  |  |
|  |  |  |  |  |  |  |
|  |  |  |  |  |  |  |
|  |  |  |  |  |  |  |
|  |  |  |  |  |  |  |
|  |  |  |  |  |  |  |
|  |  |  |  |  |  |  |
|  |  |  |  |  |  |  |

| **Baermann** |  |  |  |  |  |  |
| --- | --- | --- | --- | --- | --- | --- |
|  |  |  |  |  |  |  |
| Instructions: count larvae in sediment and differentiate | | | | |  |  |
| In the last two columns, give the number of identified larvae of both species | | | | | |  |
|  |  |  |  |  |  |  |
| **Technician:** | |  |  | **Date:** | |  |
|  |  |  |  |  |  |  |
| **ID number** | **No of larvae (sediment)** | |  | **ID number** | **No of larvae (sediment)** | |
|  | *Strongyloides* | Hookworm |  |  | *Strongyloides* | Hookworm |
|  |  |  |  |  |  |  |
|  |  |  |  |  |  |  |
|  |  |  |  |  |  |  |
|  |  |  |  |  |  |  |
|  |  |  |  |  |  |  |
|  |  |  |  |  |  |  |
|  |  |  |  |  |  |  |
|  |  |  |  |  |  |  |
|  |  |  |  |  |  |  |
|  |  |  |  |  |  |  |
|  |  |  |  |  |  |  |
|  |  |  |  |  |  |  |
|  |  |  |  |  |  |  |
|  |  |  |  |  |  |  |
|  |  |  |  |  |  |  |
|  |  |  |  |  |  |  |
|  |  |  |  |  |  |  |
|  |  |  |  |  |  |  |
|  |  |  |  |  |  |  |
|  |  |  |  |  |  |  |
|  |  |  |  |  |  |  |
|  |  |  |  |  |  |  |
|  |  |  |  |  |  |  |
|  |  |  |  |  |  |  |
|  |  |  |  |  |  |  |
|  |  |  |  |  |  |  |
|  |  |  |  |  |  |  |
|  |  |  |  |  |  |  |
|  |  |  |  |  |  |  |
|  |  |  |  |  |  |  |
|  |  |  |  |  |  |  |
|  |  |  |  |  |  |  |

**Appendix 3: Summary and grading of acute adverse events**


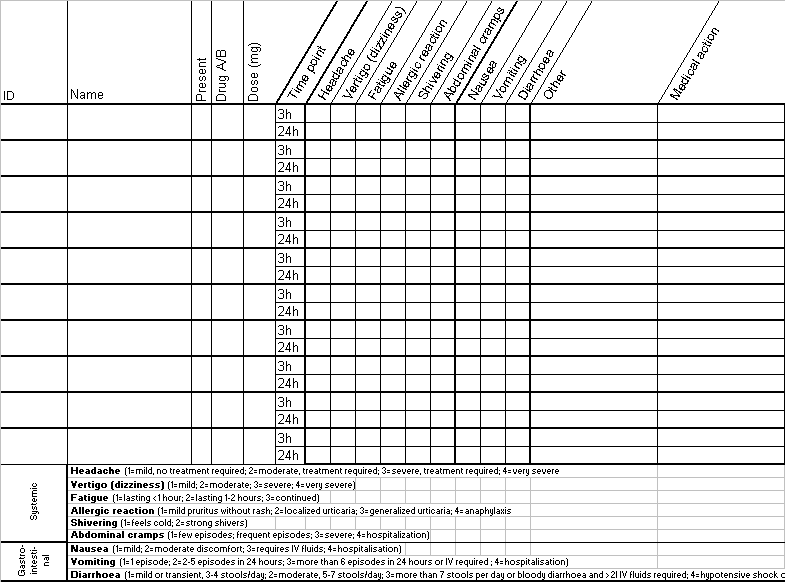

Supplement: Protocol S1 — This is the study protocol of the research reported as submitted to the Ethics Committee of the Unviversity and State of Basel (EKBB) on 3 May 2007. (0.37 MB DOC) [file pntd.0000322.s004.doc]
